# Influence of a Single Dose of Meloxicam Administrated during Canine Estrus on Progesterone Concentration and Fertility—A Clinical Case Study

**DOI:** 10.3390/ani12050655

**Published:** 2022-03-04

**Authors:** Michał Dzięcioł, Aleksandra Szpaczek, Oliwia Uchańska, Wojciech Niżański

**Affiliations:** 1 Department of Reproduction and Clinic of Farm Animals, Wroclaw University of Environmental and Life Sciences, Plac Grunwaldzki 49, 50-366 Wrocław, Poland; aleksandra.szpaczek@upwr.edu.pl (A.S.); wojciech.nizanski@upwr.edu.pl (W.N.); 2 Faculty of Veterinary Medicine, Wroclaw University of Environmental and Life Sciences, Sutent, ul. C.K. Norwida 25, 50-375 Wrocław, Poland; 110419@student.upwr.edu.pl

**Keywords:** dogs, meloxicam, progesterone, fertility, prostaglandin

## Abstract

**Simple Summary:**

The use of nonsteroidal anti-inflammatory drugs (NSAID) in medicine is mostly connected to pain relief, inflammation reduction, and treatment of fevers, but their benefits in the context of reproduction are also described in the literature. In female domestic dogs, the corpus luteum (CL) is the only source of progesterone (P4), an adequate level of which is required for the maintenance of pregnancy. Based on studies in a number of species, the use of NSAID can have both a positive and negative influence on female fertility by reducing inflammation, as well as by acting as a Cyclooxygenase 1 or 2 (COX 1 or COX 2) inhibitor and influencing the production of prostaglandins that regulate CL creation and function. In this paper, we present a case of decreased levels of progesterone measured in the peripheral blood of a female domestic dog after meloxicam administration. Our observations suggest an influence of meloxicam administration on CL function, based on decreased P4 levels. However, in contrast to observations in other species, there was no negative effect on fertility, as, despite the drop in P4 levels, the female still became pregnant and delivered puppies. This paper indicates the need for broader studies on the effect of meloxicam in canine females in the context of its safety for pregnancy (including route of administration, doses, and duration of treatment) and its effectiveness in improving fertility.

**Abstract:**

This case report presents an observation of the influence of meloxicam administration, during the periovulatory phase, on corpora lutea function in the domestic dog (Canis familiaris). A 2 year old female German Shepherd dog, with a level of progesterone suggestive of the periovulatory period (5.97 ng/mL), received a single subcutaneous injection of meloxicam (0.2 mg/kg body weight). In this female, subsequent evaluation to detect the optimal time for mating revealed a strong decrease in P4 (2.77 ng/mL), although it increased to 13.98 ng/mL within the following three days. The same female conceived and delivered a litter of the same size as in a subsequent cycle where meloxicam was not administered. The use of NSAID (nonsteroidal anti-inflammatory drugs), and particularly meloxicam, as a COX-2 inhibitor that influences the production of prostaglandins has been reported as being potentially harmful for ovulation and pregnancy in various species, including humans. In dogs, the secretion of prostaglandin E (PGE) is thought to be important for ovulation and the formation of the corpora lutea, the only source of P4, which is required for pregnancy maintenance. Although previous case reports have indicated an influence of meloxicam administration on CL function in domestic dogs, here, the decreased progesterone level observed directly after a single dose of meloxicam was only temporary, with no negative impact on fertility. Based on our observations, further studies related to the influence of the timing of meloxicam administration on ovarian cycle advancement, as well as the influence of duration of treatment, dosage, and route of administration on progesterone levels, as an indicator of CL function, are required.

## 1. Introduction

Canine reproductive physiology differs from that of other domestic animal species. Some of these differences include the duration of the luteal phase, which takes about 60 days regardless of pregnancy status, a preovulatory increase in progesterone secretion, and the lack of an acute luteolytic mechanism in the absence of pregnancy, compared to the acute prepartum luteolysis observed in pregnant animals [1,2,3]. Despite many studies on the latter issue, the mechanism of the maintenance and regression of canine CL in diestrus is still not clear [2,4]. Prostaglandin PGF2 (PGF 2), which is responsible for abrupt prepartum luteolysis, is thought not to be involved in luteolysis in non-pregnant females, but another prostaglandin (PGE), produced via cyclooxygenase-2 (COX-2) within the periovulatory follicle, is required for successful ovulation, and seems to play a supporting role in early CL stage [1,3,5,6]. Taking into account the role of cyclooxygenases in CL metabolism, the use of COX-1 and COX- 2 inhibitors in the early stages of CL formation seems to be an interesting and important issue, which needs detailed evaluation [3].

Reports related to the use of COX-2 inhibitors around ovulation, performed in other species (humans, primates, cows, rats, and rabbits), mostly suggest a significant negative effect of COX-2 inhibition on fertility, while other studies of COX-2 inhibitor administration during various stages of the ovarian cycle have reported a lack of negative impact on reproductive performance in animals [7,8,9,10,11,12,13,14,15,16,17,18,19]. Moreover, besides the use of antibiotics and hormones recommended in some cases of reduced fertility, the effects of NSAID on reducing inflammatory processes within the uterus (inhibition of prostaglandins synthesis) could be considered as potentially beneficial for improving fertility, especially in females (cows and mares) with a history of reproductive failure, or those undergoing embryo transfer procedures [20,21,22,23,24].

The measurement of progesterone levels in the peripheral blood of female domestic dogs has been used as a diagnostic tool in monitoring ovarian function, determining the optimal time for mating, and monitoring pregnancy. It allows for the detection of the preovulatory progesterone increase that is characteristic of this species, and for monitoring CL function during diestrus.

There is limited information on the effects of the use of NSAID, such as meloxicam, on canine reproductive performance, and conflicting information about their influence on bovine reproduction supports the need for further study. This paper presents a case of the use of a COX-2 inhibitor (meloxicam) during the early stage of CL formation in terms of its influence on CL function. For this purpose, we monitored progesterone levels in the peripheral blood, as well as the consequences for the fertility of the treated female.

## 2. The Case

### 2.1. Animal

The case animal, a 2 year old, 27 kg BW German Shepherd bitch, was brought to the Clinic of Reproduction to detect the most fertile period as the best time for natural mating.

According to detailed history records, the female had no health problems. She had not been mated in previous cycles due to her young age, and so while the estrus cycle ran smoothly, the level of hormones had not been monitored.

During her next heat (her third), she was brought to the Clinic to determine the optimal mating time. Progesterone levels, as well as vaginal cytology, were evaluated according to the methods described below [25,26]. Blood samples were collected by venipuncture of the cephalic vein, into anticoagulant-free tubes for serum (Separmed ^®^, CIBESMED, Basel, Switzerland). The blood samples were centrifuged, and the serum was evaluated immediately. The gradual rise in progesterone was monitored, since the vaginal cytology indicated 80% epithelial, keratinized, superficial cells.

During the second visit for progesterone evaluation (two days later), the female dog presented with an orthopedic disorder (sudden lameness), and so one dose of meloxicam (Metacam, Boehringer Ingelheim, Ingelheim am Rhein, Germany) was administered (0.2 mg/kg body weight, s.c.) (Table 1). The lameness disappeared after a single treatment, with no evidence of any of the possible side effects described by the manufacturer. Surprisingly, during the subsequent visit for estrus monitoring (performed further two days later), the level of progesterone had declined from that recorded previously, from 5.97 ng/mL to 2.77 ng/mL (Table 1). This decline was not noted in the other females monitored on the same day using the same equipment, and repeated examination of the case female, performed on the same day, confirmed a decreased level of progesterone (2.65 ng/mL). Progesterone level determinations conducted over the following days had a tendency to increase (Table 1). About 4 weeks after mating (day +30), pregnancy was confirmed, and the presence of five live fetuses was noted. Due to the unexpected change, progesterone levels were monitored throughout the pregnancy, but no further abnormalities were detected. The female delivered five healthy puppies 64 days after mating.

A complete evaluation was performed again during the next estrus cycle, including cytological examination, as well as measurement of progesterone levels [25]. No medications were administered. A continuous increase in progesterone was observed without any fluctuations (Table 1). The case animal became pregnant and gave birth to five healthy puppies 62 days after mating.

### 2.2. Progesterone Concentration Evaluation Procedure

Progesterone concentration in the peripheral blood was determined by an enzyme-linked fluorescence assay (ELFA; mini VIDAS ^®^ Biomerieux, Marcyl’Étoile France); the coefficient of variation (CV) for the mean level of progesterone 21 was 66 ng/mL at a level of 3.8% [26]. Progesterone measurement was always performed during the morning hours, both to avoid the influence of circadian variation in progesterone levels, as well as to allow a more accurate comparison of the dynamics of progesterone increase [27].

### 2.3. Retrospective Evaluation of Progesterone Behavior in Other Females

As a comparison, a retrospective analysis of the progesterone levels in other females of various breeds, aged 2 to 7 years (*n* = 35), that were patients of the same clinic within the same year, was performed, including data from the same year and measured using the same instrument. The progesterone levels of these animals did not follow a similar pattern to that of the meloxicam-treated patient; although there were fluctuations in progesterone levels when concentrations were <2 ng/mL, there was no evidence of a decrease in the progesterone level once it had passed 2 ng/mL.

## 3. Discussion

In this case, there was a decrease in peripheral blood progesterone levels after meloxicam administration (detected during routine examination two days later), which was then followed by an increase in progesterone concentration (Table 1). There was no obvious effect of the observed drop in progesterone levels on the female dog’s fertility, as she became pregnant and delivered the same number of healthy puppies (*n* = 5) during this cycle as during her subsequent pregnancy, when meloxicam was not administered. In comparison with the progesterone profiles of 35 other females, a drop in progesterone was observed only in the case study female, and it was clearly correlated with the time of meloxicam administration (Table 1). These data confirm that the first cycle evaluated was disturbed and became prolonged, which suggests that progesterone levels from the beginning of the cycle were inadequately high. However, we can surmise that, if meloxicam had not been administered, the progesterone level two days later would have allowed for successful mating on 20 November 2020, which would have resulted in conception occurring four days sooner, providing very similar results for both cycles. This conclusion seems to confirm the influence of meloxicam on the ovulation process in the first cycle.

The main indications for NSAID in medicine are for the treatment of pain, inflammation, and fever, so they are used in some orthopedic cases and as an adjunct to antibiotics and chemotherapeutics in reducing the consequences of infection [8].

The influence of the various NSAID on the fertility of male and female domestic animals has been the subject of research in many species [7,19,24,28,29,30,31,32,33]. Results obtained in humans, primates, and rabbits clearly indicate a negative effect, connected with disturbance of ovulation, leading some authors to postulate the use of meloxicam as an alternative contraceptive drug [9,10,11,13,14,18]. In women, the use of NSAID has been reported as being connected with “luteinized unruptured follicle syndrome”, which resolved when NSAID treatment was discontinued [34]. However, the administration of meloxicam at various stages of the bovine reproductive cycle showed no negative effect on reproduction [8,35,36], and was associated with improved outcomes following embryo transfer in cows, as well as in mares [20,21,23,24,30,31]. These observations, however, are in contrast with the results of Erdem [17], who found meloxicam to be harmful to pregnancy in Holstein heifers when administered during early pregnancy. It is worth mentioning that, despite the desirable anti-inflammatory effect of NSAID and the reduction in PGF2 secretion, meloxicam may have an immunosuppressive effect on nonspecific cellular and humoral responses, which, in practice, could appear as an increased sensitivity to infection [37].

Prostaglandins are one of the most important bioregulators of CL function in dogs. Various types of PGs are involved in ovulation, and support both CL function and regression. While PGF2 alpha plays a role in rapid luteolysis immediately prior to parturition, currently, PGE is believed to have a luteotropic effect during the early luteal phase [38]. Thus, PGE2 may play an important role in the regulation of progesterone secretion by the CL in female dogs [38]. Moreover, it is worth mentioning that the canine CL is the only source of progesterone, regardless of pregnancy status [6,39], and, due to the independence from gonadotropic support during the first third of diestrus, it seems to play a crucial role in the maintenance of pregnancy.

In dogs, cyclooxygenase 1 and 2 (COX-1 and COX-2) are crucial enzymes in prostaglandin biosynthesis [7,40]. The results obtained by Kowalewski et al. [40] clearly suggested that increased expression of COX-2 is associated with luteal growth and development. While the canine CL is independent of gonadotropins, prostaglandins (PGs) appear to be among its main regulators [41]. However, as the results of Tavares Pereira et al. [41] indicate, administration of a COX-2 inhibitor during early diestrus (on days 5 and 10) had little effect, and, according to the authors, could suggest the presence of strong compensatory effects in the early CL. It is thought that the timing of the administration of COX-2 inhibitors is crucial in relation to its safety for fertility.

However, despite the suggested important luteotropic role of PGE, the main luteotropic factor in the canine appears to be prolactin (PRL), which, in the period of a possible/suspected decrease of PGE levels, could also support the pregnancy by regulating placental function [2,3,6].

Due to the fact that the canine uterus is not involved in the maintenance of the CL during diestrus or pregnancy, any treatment that affects CL function, including its production of progesterone, could potentially affect fertility. Therefore, if there is a non-physiological drop in progesterone levels during diestrus, the issue of hypoluteoidism should be considered [42,43,44]. According to the literature, hypoluteoidism is characterized by the insufficient secretion of progesterone by the CL during pregnancy, which results in a failure to maintain the progesterone concentration above a critical level, and, if not detected and treated, could lead to fetal resorption or abortion [44,45,46]. The level necessary for pregnancy maintenance is considered to be above 2 ng/mL; however, in practice, when a drop in P4 to below 10 ng/mL is observed in the middle stage of pregnancy, supplementation with progestogens, such as medroxyprogesterone acetate/MPA/(which is unfortunately not free from negative side effects), altrenogest (0.088 mg/kg, PO, once daily), or progesterone in oil (1–2 mg/kg, IM, every other day), can be considered [42,47,48]. However, luteolysis can be artificially induced by dopamine agonists (which decrease the PRL level) or prostaglandin F2α; in the case of hypoluteoidism, the decreased level of progesterone occurs spontaneously [45]. Considering other drugs that can terminate pregnancy, the use of aglepristone (progesterone receptor blocker/progesterone antagonist) does not decrease progesterone levels in the peripheral blood [5,49]; an elevation of progesterone levels can even be detected, due to its greater affinity for progesterone receptors. In the context of the influence of drugs used for abortion induction in dogs upon progesterone level, it is worth mentioning that Paradis et al. [50] found that, even though PGF2 could be useful during midpregnancy, there was no significant effect on serum progesterone levels in females treated with PGF2 during the early luteal phase.

In clinical practice, hypoluteoidism used to be considered as one of the possible reasons for pregnancy loss, along with bacterial and viral infection, genetic effects, and trauma [48,51]. Hypoluteoidism can be related to a decrease in the secretion of prolactin, which is supposed to be the main luteotropic agent in dogs. Moreover, taking into account the suggested stimulating effect of relaxin (RLX) on PRL levels, insufficient relaxin secretion could cause low prolactin, which in turn would cause insufficient luteal production of progesterone [42]. It must also be mentioned that low levels of progesterone can occur due to luteolysis connected to the release of PGF 2α, secreted as a consequence of endometrial infection or inflammation [42]. According to the literature, the German Shepherd breed seems to be predisposed to hypoluteoidism [48,52]. It would seem relevant to discuss this disorder in the context of our case; however, hypoluteoidism can also be detected in other breeds, and could especially be suspected in bitches with a history of repeat pregnancy loss. In those cases, the level of P4 was monitored continuously throughout the pregnancy, and supplemented by progestogens if necessary, but the decline in progesterone levels was observed during midpregnancy and advanced pregnancy, and not during the beginning of diestrus (most commonly observed between days 20 and 35 of pregnancy) [46]. This seems to be one of the main differences between the case presented here and cases of hypoluteoidism observed and monitored in daily veterinary practice. The second difference, which indicates a direct connection between meloxicam administration and the decrease in P4 concentration, is the transitional nature of the decline in the circulating levels of progesterone. The transient decrease was detected two days after meloxicam treatment, and was then followed by increasing P4 levels, which is not observed in cases of hypoluteoidism.

According to the package insert, administration of meloxicam is contraindicated in pregnant bitches, but this is due to a lack of studies confirming its safety, rather than to any demonstrated harm to pregnancy in this species [8]. Indeed, the reduction in inflammatory processes in the uterus, which could significantly reduce fertility, seems to have a potentially beneficial effect, possibly improving fertility in those females. This could be achieved by the administration of NSAID around the time of implantation, and this would appear to warrant investigation in this species. In the case presented, the maintenance of pregnancy could suggest the safety of meloxicam use during the early metestrus period, although this cannot be confirmed due to the limited number.

## 4. Conclusions

It seems worthwhile to perform further research on how the route of meloxicam administration, dosage, and duration of treatment, as well as the timing of meloxicam administration (early or advanced pregnancy), might influence progesterone secretion and conception rate, and the course of pregnancies in treated females. In the current case, meloxicam was administered only once, and a short-duration drop in progesterone levels was observed. It would be interesting to evaluate whether the observed drop in P4 would still be temporary under continuous application, which might suggest some compensatory mechanism. Moreover, taking into account the report of Jaroszewski et al. [53] describing the influence of NSAID on progesterone production in cultured bovine luteal cells, concluding that “different NSAID can in different way influence the activity of luteal cells from corpora lutea, decreasing, being neutral or even increasing the level of produced progesterone”, the evaluation of the influence of NSAID other than meloxicam on CL function in dogs seems to be required.

## Figures and Tables

**Table 1:** Progesterone profiles in the peripheral blood in the subject female during subsequent ovarian cycles.

First Cycle												
Date	16 November 2020	18 November 2020	20 November 2020	24 November 2020	25 November 2020	27 November 2020	22 December 2020	25 December 2020	29 December 2020	30 December 2020	4 January 2021	26 January 2021
d	−6	−4	−2	+2	+3	+5	+30	+33	+37	+38	+43	+65 **
P4	3.32	5.97	2.77 *	13.98	16.54	39.09	28.13	27.91	22.73	22.61	20.04	x
Event	Estr	Estr/Mel	Estr	Estr/Mat	x	Diest	Preg5F	Preg5F	Preg5F	Preg5F	Preg5F	Part5N
						Second	cycle					
Date	22 July 2021	27 July 2021	30 July 2021	2 August 2021	3 August 2021	x	31 August 2021	x	x	x	x	2 October 2021
d	−8	−3	0	+2	+3	x	+31	x	x	x	x	+65 **
P4	1.48	1.93	5.08	19.41	32.15	x	33.51	x	x	x	x	x
Event	Estr	Estr	Estr	Estr/Mat	Diest	x	Preg5F	x	x	x	x	Part5N

d—day of cycle; P4—level of progesterone ng/mL; Event—describes the stage of the cycle (estrus/diestrus) and events such as mating or meloxicam administration. Stage of cycle described on the basis of P4 and vaginal cytology (estrus—predominance of superficial cells; diestrus—decline in the general number of cells, especially superficial cells, and reappearance of intermediate and parabasal cells); Estr—estrus; E/Mel—estrus and meloxicam administration; E/Mat—estrus and mating; Diest—diestrus; Preg5F—pregnancy detected by USG examination, confirmation of presence of five fetuses; Part5N—parturition, delivery of five newborns. * low level of progesterone was confirmed by repeated examination, to exclude the risk of false results due to technical reasons. Obtained result (2.65 ng/mL) confirmed decrease in progesterone level in case female. ** Parturition in dogs consistently occurs 65 ± 1 days after the preovulatory LH surge (day 0) in over 95% of normal pregnancies [1].

## Data Availability

The data that support the findings of this study are available from the corresponding author (M.D.), upon reasonable request.
